# Do compulsory mental health patients have a right to receive a second opinion on their treatment under Australian mental health legislation?

**DOI:** 10.1177/00048674241267219

**Published:** 2024-08-02

**Authors:** Sam Boyle, Emma Cockburn, Bianca Mandeville

**Affiliations:** 1 Australian Centre for Health Law Research, School of Law, Faculty of Business and Law, Queensland University of Technology, Brisbane, QLD, Australia; 2 School of Law, Faculty of Business and Law, Queensland University of Technology, Brisbane, QLD, Australia; 3 La Trobe Law School, La Trobe University, Melbourne, VIC, Australia

**Keywords:** Second opinion, mental health law, compulsory treatment, mental illness, legislation

## Abstract

We reviewed Australian mental health legislation to determine what obligations it places on psychiatrists to facilitate second opinions for compulsory patients who request them. Only four jurisdictions—Australian Capital Territory, Queensland, Victoria, and Western Australia—have legislated for ‘patient-initiated’ second opinions. Within these four regimes, there is variation in important aspects of the second opinion process, and there is a general absence of direction given to the second opinion providers. Based on research showing the variability of second opinion provision under New Zealand mental health legislation, we argue that this absence is likely to result in significant variation in the quality and depth of second opinions provided in Australia. We argue that New South Wales, the Northern Territory, South Australia, and Tasmania should consider formal provision for patient-initiated second opinions in their mental health legislation. We believe that such legislation ought to be aware of the barriers patients may face in accessing second opinions, and avoid exacerbating these barriers as Queensland’s legislation appears to. Also, we argue that research on current practice in Australia should be conducted to better understand the effects of legislation on second opinions, and to help determine what amounts to best practice.

Access to a second opinion is known to increase patient satisfaction with their treatment (Payne et al., 2014). It has been argued that the availability of second opinions in the context of mental health treatment should be expanded (Heuss et al., 2018) although there is little current data on how often they are used in mental health. We reviewed Australian mental health legislation to determine what obligations it places on psychiatrists to facilitate second opinions for compulsory patients who request them. We describe these as ‘patient-initiated’ second opinions to distinguish them from second opinions sought by psychiatrists. Table 1 provides a summary of the legislative provisions in Australian mental health legislation on patient-initiated second opinions for treatment. Only four jurisdictions – Australian Capital Territory (*ACT*), Queensland, Victoria and Western Australia – have legislated for the provision of patient-initiated second opinions.

**Table 1.** Provision of patient-initiated second opinions on treatment for people subject to compulsory treatment for mental illness.

Jurisdiction	Mental health legislation	Right to second opinion?	Where in legislation?	Chief Psychiatrist guidelines?
Victoria	Mental Health and Wellbeing Act 2022	Yes	ss 67-77	No
Queensland	Mental Health Act 2016	Yes	s 290	Yes
Western Australia	Mental Health Act 2014	Yes	ss 182-184	Yes
Australian Capital Territory	Mental Health Act 2015	Yes	s 15(1)(b)(i)	No
Tasmania	Mental Health Act 2013	No	-	No
South Australia	Mental Health Act 2009	No	-	No
New South Wales	Mental Health Act 2007	No	-	No
Northern Territory	Mental Health and Related Services Act 1998	No	-	No

In Victoria, the rules are contained in the mental health legislation. In Queensland and Western Australia, the rules are contained in a combination of legislation and Chief Psychiatrist guidelines. The ACT legislation simply requires that compulsory patients be informed, in writing, of their ‘right to obtain a second opinion from an appropriate mental health professional’; but no further details on this right are provided in the legislation, and no guidelines apply. The Queensland, Victoria and Western Australia regimes are broadly similar; however, they differ in some important respects, described below. The specific locations of these rules within the applicable legislation and guidelines are set out in Table S2 (see Supplemental Materials).

## When a patient-initiated second opinion may be requested

In Victoria, a person under a compulsory treatment order may seek a second opinion on treatment ‘at any time’. In Western Australia, a second opinion may be sought if the patient is ‘dissatisfied’ with their treatment. However, in Queensland, the second opinion rules only apply when the health service has been ‘unable to resolve a complaint’ made by the patient about their treatment.

## How the patient-initiated second opinion provider is selected

In Victoria, a second opinion can be sought directly by the patient from ‘any psychiatrist’. The psychiatrist may be from the same or a different mental health service, a private psychiatrist, or from the Second Psychiatric Opinion Service – an independent second opinion provider. The treating psychiatrist must ensure that reasonable supports are provided to the patient in their seeking of the second opinion.

The Queensland Guidelines stipulate that the second opinion provider be outside the patient’s treating team, but they may be from the same service. The WA Guidelines provide detailed guidance on the issue of independence of the second opinion provider. They note that various factors, including availability, timeframes and cost will be relevant to determining the appropriate provider, but that the most important factor is patient choice, after the patient has been given ‘as much relevant information as possible’. However, unlike in Victoria, the Queensland and Western Australian regimes do not provide for the patient to obtain a second opinion directly. In Queensland, the administrator of the health service is required to obtain the second opinion. In Western Australia, either the patient’s psychiatrist or the Chief Psychiatrist must obtain the second opinion. Also, unlike Victoria, Queensland and Western Australia do not have a dedicated second opinion provider.

## Process for providing a patient-initiated second opinion

All regimes require that the second opinion be provided in writing; however, there is no set form to be completed, and there is apparent discretion allowed as to the level of detail to be included. The Victorian and Western Australian legislation require a written report to be given to the treating psychiatrist and the patient. In Queensland, the provider must ‘document’ the opinion in the patient’s health records and ‘advise’ the treating team and the patient of the opinion, but the legislation does not require a report to be given to the patient.

In Victoria, if the second opinion differs from the current treatment, and the treating psychiatrist chooses not to implement it, or only implement part of it, the psychiatrist must give reasons for their decision to the patient in writing. Furthermore, the treating psychiatrist must also advise the patient of their right to apply to the Chief Psychiatrist for a review of their treatment.

In Queensland, if the second opinion differs from that of the treating psychiatrist, the second opinion provider must ‘consult with the patient’s treating team, to resolve the appropriate course of action’. If resolution does not occur, the clinical director of the medical service must be contacted for advice, and the issue may further be escalated to the Chief Psychiatrist. In Western Australia, the patient’s psychiatrist ‘must have regard to’ the second opinion; however, the legislation does not prescribe a process to follow if there is disagreement. The patient, if they remain dissatisfied, may request a third opinion; however, the treating psychiatrist or Chief Psychiatrist have discretion as to whether to facilitate this. Otherwise, if the patient remains dissatisfied with their treatment, they may seek resolution from the Chief Psychiatrist.

## Discussion

The above shows that only half of the Australian jurisdictions have any applicable law on patient-initiated second opinions for people under compulsory treatment, and within those that do, there is considerable variety in process and prescriptiveness. Dawson et al. (2015) examined practices in New Zealand regarding the provision of second opinions, which are mandatory (and thus, not patient-initiated) at the commencement of compulsory mental health orders and for authorisation of electroconvulsive therapy. Like the Australian legislation, the New Zealand scheme did not have set forms for these second opinions, and individual medical centres developed their own practices. Dawson et al. found that the reports created by the second opinion providers ranged from very detailed to very brief, with one report containing only a single word, which was the suggested medication. This suggests that in the absence of specific requirements under Australian mental health legislation for what a second opinion provider must do, there is likely to be significant variation in the quality and depth of second opinions provided. Making second opinions available to patients receiving compulsory care has cost and time implications for both the treating teams and the psychiatrists providing the second opinions. In this context, clarity regarding a treating team’s obligations is important. In the ACT, and in jurisdictions with no formal provision for second opinions at all, namely, New South Wales, Northern Territory, South Australia and Tasmania, there is potential for even greater inconsistency in practice.

Also, requesting a second opinion may be daunting for a compulsory mental health patient. Individuals with mental disabilities may have difficulties articulating dissatisfaction with their treatment (Heuss et al., 2018). Furthermore, mental health patients may believe that a request for a second opinion would be perceived as questioning their treating psychiatrist’s decisions. In Victoria, which has the most facilitative regime for patient-initiated second opinions, the Royal Commission into that state’s mental health system heard that patients still had difficulties in accessing second opinions (Royal Commission into Victoria’s Mental Health System, 2021, vol. 4: 401). These findings relate to the previous *Mental Health Act 2014* (Vic); however, the regime for second opinions under that legislation was largely equivalent to the current legislation. The Royal Commission heard that difficulties were caused by several barriers, including the power imbalance between patient and treating team. We believe that the Queensland regime, which requires that patients make a complaint about their treatment before a second opinion can be accessed, may exacerbate these difficulties.

We argue that mental health legislation in New South Wales, the Northern Territory, South Australia and Tasmania should consider developing formal provision for patient-initiated second opinions on treatment in order to clarify the medical providers’ obligations in this regard. We believe that this legislation ought to be aware of the barriers patients may face in accessing them, and avoid exacerbating the problem as Queensland’s legislation appears to. However, we note that there is currently a lack of quantitative data on patient-initiated second opinion regimes in Australia, and a lack of qualitative analysis of the kind performed in New Zealand by Dawson et al. Without this data, we cannot be certain what effect legislation has on practice. Therefore, we argue that this research should be performed in Australia. Such research could also support a greater understanding of what constitutes best practice in this area.

## Supplemental Material

sj-pdf-1-anp-10.1177_00048674241267219 – Supplemental material for Do compulsory mental health patients have a right to receive a second opinion on their treatment under Australian mental health legislation? Supplemental material, sj-pdf-1-anp-10.1177_00048674241267219 for Do compulsory mental health patients have a right to receive a second opinion on their treatment under Australian mental health legislation? by Sam Boyle, Emma Cockburn and Bianca Mandeville in Australian & New Zealand Journal of Psychiatry
