# Supplementary material for: Do compulsory mental health patients have a right to receive a second opinion on their treatment under Australian mental health legislation?
Source: Aust N Z J Psychiatry. 2024 Aug 2;58(11):927–9. doi: 10.1177/00048674241267219 (PMC11497731; doi:10.1177/00048674241267219)
Supplement: sj-pdf-1-anp-10.1177_00048674241267219 – Supplemental material for Do compulsory mental health patients have a right to receive a second opinion on their treatment under Australian mental health legislation? [file sj-pdf-1-anp-10.1177_00048674241267219.pdf]

Table 2 – Guide to Australian legislation and guidelines on second opinions on treatment for people subject to compulsory treatment for mental illness

Jurisdiction	Legislation/ guidelines	Who may request a second opinion	When a second opinion may be requested	Obligation to assist patient?	Identity of second opinion provider	Obligations on second opinion provider
**Victoria**	Mental Health and Wellbeing Act 2022	s 67(2)	s 67(1)	s 67(3)	s 68	ss 69, 70, 72, 73, 74
**Queensland**	Mental Health Act 2016	s 290(2), Sch 3 Dictionary (definition 'interested person')	s 290(1)	Not stated	s 290(3)(a)	Not stated
	Chief Psychiatrist Guidelines	Not stated	2.1	Not stated	2.4	2.5, 2.6
**Western Australia**	Mental Health Act 2014	s 182(1)	s 182(2)	Not stated	s 182(5)	ss 79(1), 80(1), 182(6), 182(7)
	Chief Psychiatrist Guidelines	Not stated	Not stated	Not stated	Guideline (c)	Guideline (d)
**ACT**	Mental Health Act 2015	s 15(1)(b)(i)	Not stated	Not stated	Not stated	Not stated
